# The Activity of the *Pseudomonas aeruginosa* Virulence Regulator σ^VreI^ Is Modulated by the Anti-σ Factor VreR and the Transcription Factor PhoB

**DOI:** 10.3389/fmicb.2016.01159

**Published:** 2016-08-03

**Authors:** Jose M. Quesada, Joaquín R. Otero-Asman, Karlijn C. Bastiaansen, Cristina Civantos, María A. Llamas

**Affiliations:** ^1^Department of Environmental Protection, Estación Experimental del Zaidín-Consejo Superior de Investigaciones CientíficasGranada, Spain; ^2^Section of Molecular Microbiology, Department of Molecular Cell Biology, VU University AmsterdamAmsterdam, Netherlands

**Keywords:** *Pseudomonas aeruginosa*, gene regulation, signal transduction, extracytoplasmic function sigma factor, phosphate starvation, PhoB

## Abstract

Gene regulation in bacteria is primarily controlled at the level of transcription initiation by modifying the affinity of the RNA polymerase (RNAP) for the promoter. This control often occurs through the substitution of the RNAP sigma (σ) subunit. Next to the primary σ factor, most bacteria contain a variable number of alternative σ factors of which the extracytoplasmic function group (σ^ECF^) is predominant. *Pseudomonas aeruginosa* contains nineteen σ^ECF^, including the virulence regulator σ^VreI^. σ^VreI^ is encoded by the *vreAIR* operon, which also encodes a receptor-like protein (VreA) and an anti-σ factor (VreR). These three proteins form a signal transduction pathway known as PUMA3, which controls expression of *P. aeruginosa* virulence functions. Expression of the *vreAIR* operon occurs under inorganic phosphate (Pi) limitation and requires the PhoB transcription factor. Intriguingly, the genes of the σ^VreI^ regulon are also expressed in low Pi despite the fact that the σ^VreI^ repressor, the anti-σ factor VreR, is also produced in this condition. Here we show that although σ^VreI^ is partially active under Pi starvation, maximal transcription of the σ^VreI^ regulon genes requires the removal of VreR. This strongly suggests that an extra signal, probably host-derived, is required *in vivo* for full σ^VreI^ activation. Furthermore, we demonstrate that the activity of σ^VreI^ is modulated not only by VreR but also by the transcription factor PhoB. Presence of this regulator is an absolute requirement for σ^VreI^ to complex the DNA and initiate transcription of the PUMA3 regulon. The potential DNA binding sites of these two proteins, which include a *pho box* and −10 and −35 elements, are proposed.

## Introduction

Regulation of gene expression allows bacteria to adapt rapidly to alterations in their environment. This regulation occurs primarily at the level of transcription initiation by modifying promoter recognition of the RNA polymerase (RNAP) holoenzyme. The RNAP holoenzyme of bacteria comprises a five-subunit core enzyme (RNAPc; subunit composition α2ββ′ω) and a dissociable sigma (σ) subunit (Murakami and Darst, 2003). The σ factor contains most promoter recognition determinants and confers promoter specificity to the RNAP. All bacteria contain a primary σ factor (i.e., σ^70^) that recognizes similar target promoter sequences and controls expression of genes required for general functions. Promoter recognition by σ^70^ is often modulated by transcription factors that either enhance or inhibit such recognition and therefore gene transcription (Ishihama, 2000; Martinez-Antonio et al., 2006). In addition, most bacteria contain several alternative σ factors that recognize alternative promoter sequences and activate expression of functions required only under specific circumstances (Ishihama, 2000). Therefore, the promoter recognition of the RNAP is modulated first by substitution of the σ subunit and secondly by the interaction with transcription factors.

The largest and most diverse group of bacterial alternative σ factors is the Group IV, which consists of the so-called extracytoplasmic function (ECF) σ factors (σ^ECF^). These σ factors control expression of important bacterial functions such as stress responses, iron uptake and pathogenicity (Lonetto et al., 1994; Helmann, 2002; Bastiaansen et al., 2012; Mascher, 2013). Both expression and activation of σ^ECF^ are tightly regulated processes that usually occur in response to environmental signals. The post-translational control of σ^ECF^ is carried out by anti-σ factors that bind to and sequester the σ^ECF^, which is only released and activated in the presence of an inducing signal. The functional unit of the σ^ECF^-dependent signaling is therefore formed by the σ^ECF^ and its cognate anti-σ factor, and the genes encoding these two proteins are normally co-transcribed. This signal transduction cascade resembles that of the two-component systems in which a membrane bound histidine kinase controls the activity of a transcription factor (known as response regulator) that also mediates a cellular response through differential expression of target genes (Stock et al., 2000). However, whereas activation of two-components system involves phosphotransfer reactions, liberation and activation of the σ^ECF^ in response to the inducing signal requires the targeted proteolysis of the anti-σ factor (Qiu et al., 2007; Ades, 2008; Draper et al., 2011; Bastiaansen et al., 2014, 2015).

**Figure 1:**
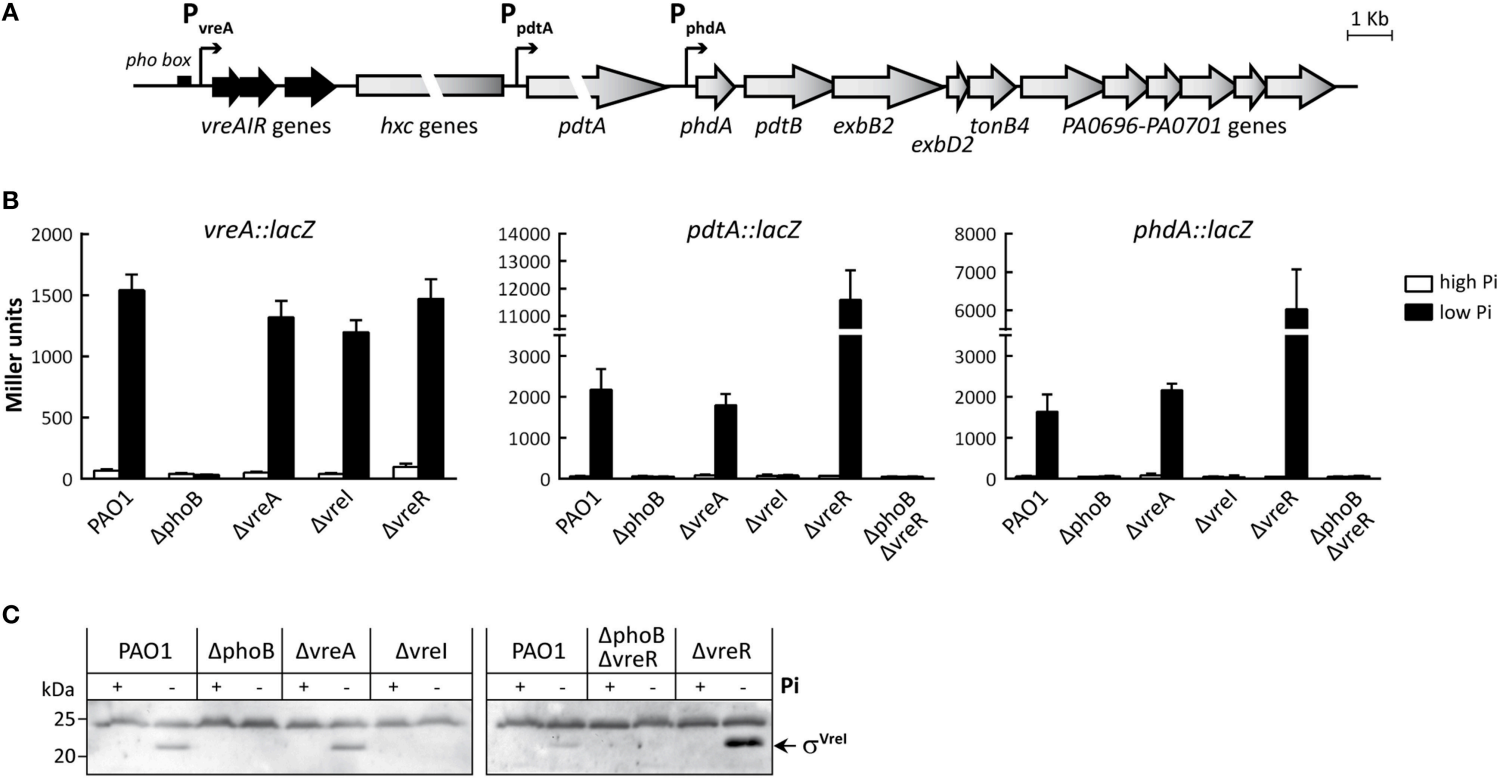
**Genetic organization and expression of the PUMA3 regulon. (A)** Transcriptional organization of the *vreAIR* locus encoding the PUMA3 CSS system (black) and the downstream PUMA3-regulated genes (gray). The big arrows represent the different genes, their relative sizes, and their transcriptional orientation. Below each arrow, the name of the gene, gene cluster or the PA number (http://www.pseudomonas.com) is indicated. Small arrows represent the promoters identified within the PUMA3 system (Llamas et al., 2009; Faure et al., 2013). The *pho box* present in the *vreA* promoter (Faure et al., 2013) is also indicated. **(B)** β-galactosidase activity of the *P. aeruginosa* PAO1 wild-type strain and the indicated mutants bearing the pMP220-derivated plasmids containing the indicated *lacZ* fusion (Table 1) after 18 h of growth in high or low Pi conditions. **(C)** Detection of σ^VreI^ in *P. aeruginosa* PAO1 wild-type strain and the indicated mutants. Proteins were detected by Western-blot using a polyclonal anti-VreI antibody. The positions of the molecular size marker (in kDa) and of the σ^VreI^ protein are shown.

A high number of σ^ECF^ in a bacterial genome usually reflects the diversity of the bacterial living environment (Staroń et al., 2009). The human opportunistic pathogen *Pseudomonas aeruginosa*, which thrives in diverse habitats ranging from soil to the human airways, encodes nineteen σ^ECF^ (Visca et al., 2002; Llamas et al., 2008, 2014). Most *P. aeruginosa* σ^ECF^ belong to the iron-starvation group (Leoni et al., 2000) and initiate transcription of iron uptake functions. Expression of these σ factors is usually regulated by iron through the ferric-uptake regulator (Fur) repressor, and their function is normally activated by an iron carrier (i.e., siderophore) via a regulatory pathway known as cell-surface signaling (CSS) (Llamas et al., 2014). Apart from the σ^ECF^/anti-σ factor pair, the CSS cascade also involves an outer membrane receptor of the TonB-dependent family (Llamas et al., 2014). CSS receptors usually have a dual function: transduce the presence of the signal to the anti-σ factor which activates the σ^ECF^ in the cytosol, and mediate the uptake of the inducing signal (i.e., siderophore) (Llamas et al., 2014). Moreover, *P. aeruginosa* contains two CSS σ factors that control expression of virulence genes (Llamas et al., 2014). This includes σ^PvdS^, which responds to *P. aeruginosa'*s own siderophore pyoverdine and regulates the production of exotoxin A (*toxA*) and PrpL endoprotease (*prpL*) (Lamont et al., 2002). The second example is σ^VreI^, which regulates expression of several potential virulence factors, including secreted proteins and secretion systems (Figure 1A), and induces *P. aeruginosa* virulence (Llamas et al., 2009). σ^VreI^ is encoded by the second gene of the *vreAIR* operon, which also encodes a CSS-like receptor (VreA) and an anti-σ factor (VreR) (Llamas et al., 2009). These three proteins form the PUMA3 CSS system (Llamas et al., 2009, 2014). This system has a number of features that differentiate it from most CSS systems. First, the CSS receptor VreA lacks the C-terminal β-barrel domain typical of TonB-dependent receptors and seems to be located in the periplasm instead of in the outer membrane (Llamas et al., 2009). This suggests that this protein is only involved in signal transduction and not in the uptake of the signal molecule. In addition, expression of the *vreAIR* operon is not regulated by iron and Fur but by phosphate (Pi) and the PhoB transcription factor (Faure et al., 2013). In *P. aeruginosa*, the level of Pi in the environment is sensed by the phosphate-specific ABC transport Pst system, which under Pi starvation conditions mediates Pi transport and activates the PhoR-PhoB two-component system (Lamarche et al., 2008). Upon activation, the PhoR histidine kinase promotes phosphorylation of its cognate DNA-binding response regulator PhoB. Phosphorylated PhoB controls the expression of a large set of genes by binding as a dimer to a *pho box*, a 22-bp specific DNA sequence in the promoter region of the PhoB regulon genes (Blanco et al., 2002). Interestingly, the genes belonging to the PUMA3 regulon are also expressed in response to Pi starvation in a σ^VreI^-dependent manner (Faure et al., 2013). This is an intriguing observation since in this condition the genes encoding both σ^VreI^ and its cognate repressor, the VreR anti-σ factor, are expressed. This study was conducted to elucidate how the activity of σ^VreI^ is modulated in Pi starvation conditions.

## Materials and methods

### Bacterial strains and growth conditions

Strains used in this study are listed in Table 1. Bacteria were grown in liquid LB (Sambrook et al., 1989) or in 0.3% (w/v) proteose peptone (DIFCO) containing 100 mM HEPES, 20 mM NH_4_Cl, 20 mM KCl, 3.2 mM MgCl_2_, and 0.4% (w/v) glucose (pH 7.2), without (low Pi) or with 10 mM KH_2_PO_4_ (high Pi), on a rotatory shaker at 37°C and 200 rpm. When required, 1 mM isopropyl β-D-1-thiogalactopyranoside (IPTG) was added to the medium to induce full expression from the pMMB67EH P*tac* promoter. Antibiotics were used at the following final concentrations (μg ml^−1^): ampicillin (Ap), 100; gentamicin (Gm), 20; kanamycin (Km), 50; nalidixic acid (Nal), 10; piperacillin (Pip), 25; rifampicin (Rif) 10; streptomycin (Sm), 100; tetracycline (Tc), 20.

**Table 1:** **Bacterial strains and plasmids used in this study^**a**^**.

**Strains or plasmid**	**Relevant characteristics**	**References**
***E. coli***		
BL21 (DE3)	F^−^*lon ompT hsdS* (^−^r_*B*_ m^−^_*B*_) *gal dcm* λ(DE3)	Jeong et al., 2009
CC118λ*pir*	Δ(*ara-leu*) *araD ΔlacX74 galE galK phoA20 thi-1 rpsE rpoB argE recA1*, lysogenized with λ*pir*; Rif^R^	Herrero et al., 1990
DH5α	*supE44 Δ*(*lacZYA-argF*)*U169* ϕ80 *lacZ*DM15 *hsdR17* (r*K*− m*K*+) *recA1 endA1 gyrA96 thi1 relA1*; Nal^R^	Hanahan, 1983
***P. aeruginosa***		
PAO1	Wild-type strain	Jacobs et al., 2003
ΔphoB	Markerless PAO1 null mutant in the *phoB* (PA5360) gene	Faure et al., 2013
ΔvreA	Markerless PAO1 null mutant in the *vreA* (PA0674) gene	This study
ΔvreI	Markerless PAO1 null mutant in the *vreI* (PA0675) gene	Faure et al., 2013
ΔvreR	Markerless PAO1 null mutant in the *vreR* (PA0676) gene	This study
ΔphoB ΔvreR	Markerless PAO1 double null mutant in the *phoB* and *vreR* genes	This study
**PLASMIDS**		
pBBR1MCS-5	Broad-host range plasmid, *ori*TRK2; Gm^R^	Kovach et al., 1995
pBBRvreR	pBBR1MCS-5 carrying in KpnI-HindIII a 0.96-Kb PCR fragment containing the entire *P. aeruginosa vreR* (PA0676) gene; Gm^R^	This study
pCR2.1-TOPO	TA cloning vector for the direct ligation of PCR products; Ap^R^, Km^R^	Invitrogen
pTOPO-Pr0690	pCR2.1-TOPO carrying the *P. aeruginosa pdtA* (PA0690) promoter amplified by PCR from the pMP0690 plasmid; Ap^R^, Km^R^	This study
pTOPO-Pr0691b	pCR2.1-TOPO carrying the *P. aeruginosa phdA* (PA0691) promoter amplified by PCR from the pMP0691b plasmid; Ap^R^, Km^R^	This study
pET28b(+)	Translation vector for cloning and expressing recombinant proteins in *E. coli*. Contains a 6xHis fusion tag; Km^R^	Novagen
pET-phoB	pET28b(+) carrying in NdeI-BamHI a 0.69-Kb PCR fragment containing the *P. aeruginosa phoB* (PA5360) gene downstream a 6xHis tag; Km^R^	This study
pET-vreI	pET28b(+) carrying in NdeI-BamHI a 0.56-Kb PCR fragment containing the *P. aeruginosa vreI* (PA0675) gene downstream a 6xHis tag; Km^R^	This study
pKNG101	Gene replacement suicide vector, *ori*R6K, *oriT*RK2, *sacB;* Sm^R^	Kaniga et al., 1991
pKΔvreA	pKNG101 carrying in XbaI-BamHI a 2.7-Kb PCR fragment containing the regions up- and downstream the *P. aeruginosa vreA* (PA0674) gene; Sm^R^	This study
pKΔvreR	pKNG101 carrying in XbaI-BamHI a 2.05-Kb PCR fragment containing the regions up- and downstream the *P. aeruginosa vreR* (PA0676) gene; Sm^R^	This study
pMMB67EH	IncQ broad-host range plasmid, *lacI^q^*; Ap^R^	Fürste et al., 1986
pMMBphoB	pMMB67EH carrying in EcoRI-HindIII a 0.8 Kb PCR fragment containing the *P. aeruginosa phoB* (PA5360) gene; Ap^R^	This study
pMMB-VreR	pMMB67EH carrying in KpnI-HindIII a 0.96-Kb PCR fragment containing the entire *P. aeruginosa vreR* (PA0676) gene; Ap^R^	This study
pMMB/VreR-HA	pMMB67EH carrying in EcoRI-XbaI a 0.99-Kb PCR fragment containing a C-terminally HA-tagged *P. aeruginosa vreR* gene; Ap^R^	This study
pMMB-VreR43	pMMB67EH carrying in KpnI-HindIII a 0.13-Kb PCR fragment encoding the first 43 amino acids of the P. *aeruginosa vreR* gene; Ap^R^	This study
pMMB-VreR86	pMMB67EH carrying in KpnI-HindIII a 0.26-Kb PCR fragment encoding the first 86 amino acids of the *P. aeruginosa vreR* gene; Ap^R^	This study
pMMB-VreR110	pMMB67EH carrying in KpnI-HindIII a 0.33-Kb PCR fragment encoding the first 110 amino acids of the *P. aeruginosa vreR* gene; Ap^R^	This study
pMUM3	pMMB67EH carrying the *vreI* (PA0675) gene; Ap^R^	Llamas et al., 2009
pMP220	IncP broad-host-range *lacZ* fusion vector; Tc^R^	Spaink et al., 1987
pMP0690	pMP220 carrying in EcoRI-BamHI a 0.53-Kb PCR fragment containing the *P. aeruginosa pdtA* (PA0690) promoter (*pdtA::lacZ* transcriptional fusion); Tc^R^	This study
pMP0691b	pMP220 containing the *phdA::lacZ* transcriptional fusion; Tc^R^	Llamas et al., 2009
pMPR3	pMP220 containing the *vreA::lacZ* transcriptional fusion; Tc^R^	Faure et al., 2013

a *Ap^R^, Cm^R^, Gm^R^, Km^R^, Nal^R^, Rif^R^, Sm^R^ and Tc^R^, resistance to ampicillin, chloramphenicol, gentamicin, kanamycin, nalidixic acid, rifampicin, streptomycin and tetracycline, respectively*.

### Plasmids construction and molecular biology

Plasmids used are described in Table 1 and primers listed in Table S1. PCR amplifications were performed using Phusion® Hot Start High-Fidelity DNA Polymerase (Finnzymes) or Expand High Fidelity DNA polymerase (Roche). Nucleotide substitutions or deletions in the *pdtA* and *phdA* promoters were generated by whole plasmid PCR site-directed mutagenesis (Fisher and Pei, 1997) with a pair of complementary mutagenic primers using the pTOPO-Pr0690 and pTOPO-Pr0691b plasmids (Table 1), respectively, as templates. After mutagenesis, the promoters were subcloned in the pMP220 plasmid as EcoRI-XbaI (P_pdtA_) or BglII-KpnI (P_*phdA*_) restriction fragments. All constructs were confirmed by DNA sequencing and transferred to *P. aeruginosa* by electroporation (Choi et al., 2006). Construction of null mutants was performed by allelic exchange using the suicide vector pKNG101 as described before (Bastiaansen et al., 2014). Southern blot analyses to confirm the chromosomal gene deletion were performed as described (Llamas et al., 2000).

### Enzyme assay

β-galactosidase activities in soluble cell extracts were determined using *o*-nitrophenyl-b-D-galactopyranoside (ONPG) (Sigma-Aldrich) as described before (Llamas et al., 2006). Each condition was tested in duplicate in at least three biologically independent experiments and the data given are the average with error bars representing standard deviation (SD). Activity is expressed in Miller units.

### Production of α-VreI and α-VreR antibodies

To obtain relatively pure protein recombinant, VreI and VreR were expressed as an insoluble protein in *E. coli* TOP10F' using an aggregation tag. Inclusion bodies were isolated as followed: bacterial cells were resuspended in 5 ml solution buffer (50 mM Tris-Hcl, 25% sucrose, 1 mM NaEDTA, 10 mM DTT, 0.4 mg/ml lysozyme, 20 μg/ml DNAse I and 2 mM MgCl_2_). Following sonication, 5 ml lysis buffer was added (50 mM Tris-HCl, 1% Triton X-100, 1% Na deoxycholate, 100 mM NaCl, 10 mM DTT) and the suspension was incubated on ice for 1 h. After a snap freezing and thawing cycle the total amount of NaEDTA and MgCl_2_ was increased to 15 mM and 6 mM, respectively. Inclusion bodies were pelleted at 11.000 × g for 20 min at 4°C and washed with a buffer containing 50 mM Tris-HCl, 1% Triton X-100, 100 mM NaCl, 1 mM NaEDTA and 1 mM DTT. Following sonication to obtain a homogenous suspension and another centrifugation step, washing was performed in the same buffer omitting Triton X-100. Subsequently, inclusion bodies were boiled in SDS-PAGE sample buffer. Proteins were analyzed by SDS-PAGE containing 12% (w/v) acrylamide and the VreI and VreR proteins were excised from the gel following an imidazole-zinc staining. The proteins were electroeluted out of the gel and purified VreI and VreR were sent to Innovagen (Sweden) for antibody production. Rabbits were immunized at day 0 and subsequently given boosters at days 14, 28, 49, and 70. At day 84 rabbits were sacrificed and serum was isolated. Prior Western-blot, serum was concentrated using 30K centrifugal filter units (Millipore) at 4000 rpm for 15 min.

### SDS-PAGE and Western-blot

Bacteria were grown until late log phase and pelleted by centrifugation. Samples were normalized according to the OD_660_ of the culture, solubilized in Laemmli buffer and heated for 10 min at 95°C. Proteins were separated by SDS-PAGE containing 12 or 15% (w/v) acrylamide and electrotransferred to nitrocellulose membranes. Ponceau S staining was performed as a loading control. Immunodetection was realized using polyclonal antibodies directed against the σ^VreI^ or the VreR proteins, or a monoclonal antibody directed against the influenza hemagglutinin epitope (HA.11, Covance). The second antibody, either the horseradish peroxidase-conjugated goat anti-rabbit IgG (Sigma-Aldrich) or the horseradish peroxidase-conjugated rabbit anti-mouse (DAKO), was detected using the SuperSignal® West Femto Chemiluminescent Substrate (Thermo Scientific). Blots were scanned and analyzed using the Quantity One version 4.6.7 (Bio-Rad).

### RNA preparation

*P. aeruginosa* cells were grown until late exponential phase in low or high phosphate medium. Total bacterial RNA was isolated by the hot phenol method using the TRI® Reagent protocol (Ambion) as described before (Llamas et al., 2008). RNA quantity and quality was assessed by UV absorption at 260 nm in a ND-1000 Spectrophotometer (NanoDrop Technologies, USA).

### 5′ RACE

The transcription start points were determined using the 5′ RACE System for Rapid Amplification of cDNA Ends (Invitrogen). RNA isolated from *P. aeruginosa* PAO1 or ΔvreR cells grown in low Pi was used as the template for 5′ RACE analysis. The primers used in this analysis are shown in Table S1. The 5′ RACE reactions were performed as recommended by the manufacturer and analyzed by agarose gel electrophoresis to assess purity and product size. Single cDNA bands were obtained for the reactions and, upon purification, were sequenced using a nested gene-specific primer to locate the 5′ end of the transcript. The sequencing results of the 5′ RACE product were aligned with the *P. aeruginosa* PAO1 genome sequence.

### Primer extension analysis

Primer extension analyses were done basically as described by Marques et al. (1994) using 12 μg of total RNA in each reaction. About 10^5^ cpm of [γ-^32^P]-labeled 5′-end oligonucleotides (Table S1) was used as primers in extension reactions. The cDNA products obtained after the reverse transcriptase reaction were separated and analyzed in urea-polyacrylamide sequencing gels. Visualization of the gels was performed using the Fujifilm imaging plate BAS-MS 2040.

### PhoB and σ^VreI^ protein purification

His-tagged PhoB and σ^VreI^ proteins were produced in *E. coli* BL21 from the pET-phoB and pET-vreI plasmids, respectively, and purified by affinity chromatography. Cells were grown overnight at 18°C in LB supplemented with 0.1 mM IPTG and harvested by centrifugation. The pellet was resuspended in 30 ml of buffer A (20 mM Tris-HCl, 0.1 mM EDTA, 300 mM NaCl, 5% glycerol, 10 mM imidazole, 5 mM β-mercaptoethanol; pH 7.25) supplemented with 1x Complete protease inhibitor cocktail (Roche) and broken by repeated French Press passages at 1000 psi. Following centrifugation at 20.000 × g for 1 h the soluble fraction was passed through a 0.22 μm filter (Millipore) and loaded onto a 5 ml HisTrapHP chelating column (GE Healthcare) previously equilibrated in buffer A. PhoB and σ^VreI^ were eluted with a 10 mM to 500 mM imidazole gradient in buffer A and dialyzed against buffer B (50 mM Tris-HCl pH 7.5, 10 mM MgCl_2_, 1 mM DTT).

### Electrophoretic mobility shift assays

Two different EMSA methods were used in this work, the classic method using radioactive labeled DNA (Rojas et al., 2003) and a new method using fluorescein labeled DNA (Blanco et al., 2011). In both methods, phosphorylated PhoB protein was used. The protein was phosphorylated in 50 mM Tris-HCl, 10 mM MgCl_2_, 1 mM DTT, and 9 mM acetylphosphate reaction buffer at 37°C for 60 min as previously described (McCleary, 1996). For the radioactive method, dsDNA probe containing the promoter region of the *P. aeruginosa pdtA* gene was obtained by annealing non-labeled complementary oligonucleotides (Table S2). The *pstC* and *fiuA* promoter regions were amplified by PCR using genomic DNA from *P. aeruginosa* PAO1. These DNA fragments were then end-labeled with [γ-^32^P] deoxyadenosine triphosphate (ATP) using the T4 polynucleotide kinase. A 10 μl sample containing 0.002 pmols of labeled DNA (1.5 × 10^4^ cpm) was incubated with increasing concentrations of phosphorylated PhoB and/or σ^VreI^ proteins for 20 min in binding buffer (12 mM Tris-HCl, pH 7, 23.6 mM NaCl, 0.12 M magnesium acetate, 0.24 mM EDTA, 0.24 mM DTT, 1.2% [v/v] glycerol, and 2.3 mM acetylphosphate) containing 20 μg/ml of polyd(IC) and 200 μg/ml of bovine serum albumin (BSA). DNA-protein complexes were resolved by electrophoresis on 4% (w/v) non-denaturing polyacrylamide gels in Tris/Glycine buffer (512 mM Tris, 58.6 mM glycine). For the second method, a 5′ end fluorescein-labeled oligonucleotide was annealed with the complementary strand (Table S2) to obtain dsDNA. In a final volume of 10 μl EMSA samples contained 0.025 nmols of fluorescein-labeled dsDNA and variable amounts of purified PhoB and/or σ^VreI^ proteins were incubated in the same binding buffer described above containing polyd(IC) but not BSA during 20 min at 37°C. In the competition experiment, increasing amounts of an unlabeled competitor dsDNA was added to the EMSA reaction. Samples were loaded onto 8% non-denaturing polyacrylamide gels prepared in Tris/Glycine buffer and run at 50 V at room temperature. The fluorescence signal was detected on a conventional UV transilluminator and pictures were taken with the gel-recoding apparatus Minilumi bio-imaging system (Bio-Imaging Systems Ltd).

### Computer-assisted analyses

Sequence analyses of the *Pseudomonas* genomes were performed at http://www.pseudomonas.com (Winsor et al., 2011) and sequence alignments with ClustalW (Thompson et al., 1994).

## Results

### Effect of the PUMA3 proteins on the expression of the *vreAIR* gene cluster and the σ^VreI^-regulated genes under Pi starvation

To assess the expression of the PUMA3 genes, we used *lacZ* transcriptional fusions to three PUMA3 promoters: P_vreA_, P_pdtA_, and P_phdA_ (Figure 1A). P_vreA_ is the promoter of the *vreAIR* operon, P_pdtA_ transcribes solely the *pdtA* gene, and P_phdA_ transcribes the *phdA, pdtB, exbB2D2, tonB4*, and PA0696-PA0701 genes (Figure 1A; Faure et al., 2013). Activity of these three promoters was tested by β-galactosidase assay in the *P. aeruginosa* PAO1 wild-type strain and in the PUMA3 deletion mutants ΔvreA, ΔvreI, and ΔvreR upon growth in low and high Pi conditions. A ΔphoB mutant was also included in the assay. The P_vreA_ was active in low Pi in a PhoB-dependent manner (Figure 1B), as reported previously (Faure et al., 2013). In the ΔvreA, ΔvreI and ΔvreR mutants this promoter reached wild-type levels (Figure 1B), showing that the PUMA3 CSS system is not involved in the regulation of its own expression. The PUMA3-regulated promoters P_pdtA_ and P_phdA_ were also active in low Pi and both the PhoB and σ^VreI^ proteins were required for such activation (Figure 1B). However, the activity of these promoters in the ΔvreA mutant reached wild-type levels (Figure 1B), which indicates that the VreA receptor is not involved in the expression of the PUMA3 regulon under Pi starvation. Expression from P_pdtA_ and P_phdA_ correlates with σ^VreI^ production, which occurs in the wild-type PAO1 and ΔvreA strains upon growth in low Pi but not in high Pi and does not occur in the ΔphoB mutant (Figure 1C). This confirms that PhoB is required for σ^VreI^ production. Interestingly, in the ΔvreR mutant the activity of P_pdtA_ and P_phdA_ in low Pi was considerably higher than in the PAO1 wild-type strain (5.3- and 3.7-fold higher, respectively) (Figure 1B). This indicates that full activation of σ^VreI^ requires the removal of VreR, which verifies the anti-σ role of this protein. In accordance, the amount of σ^VreI^ in the ΔvreR mutant was considerably higher than in the PAO1 wild-type strain (Figure 1C,-Pi). This supports previous results showing that an HA-tagged version of the σ^VreI^ protein is more stable in the absence of the anti-σ factor VreR (Llamas et al., 2009), and suggests that VreR promotes σ^VreI^ degradation. The high *pdtA* and *phdA* expression observed in the ΔvreR mutant was PhoB- and σ^VreI^-dependent since activity of the *lacZ* fusions was completely abolished in a ΔphoB ΔvreR double mutant (Figure 1B) that lacks PhoB and in which σ^VreI^ is not produced (Figure 1C). Activity of the three promoters in the *phoB* mutants could be complemented by providing the *phoB* gene *in trans* (Figure S1). Complementation was only partial (~35–55% of the activity in wild-type conditions) since overproduction of PhoB from plasmid slightly diminished promoter activities, as observed in the PAO1 wild-type strain (Figure S1).

### Role of the N-terminus of VreR in the regulation of σ^VreI^ activity

**Figure 2:**
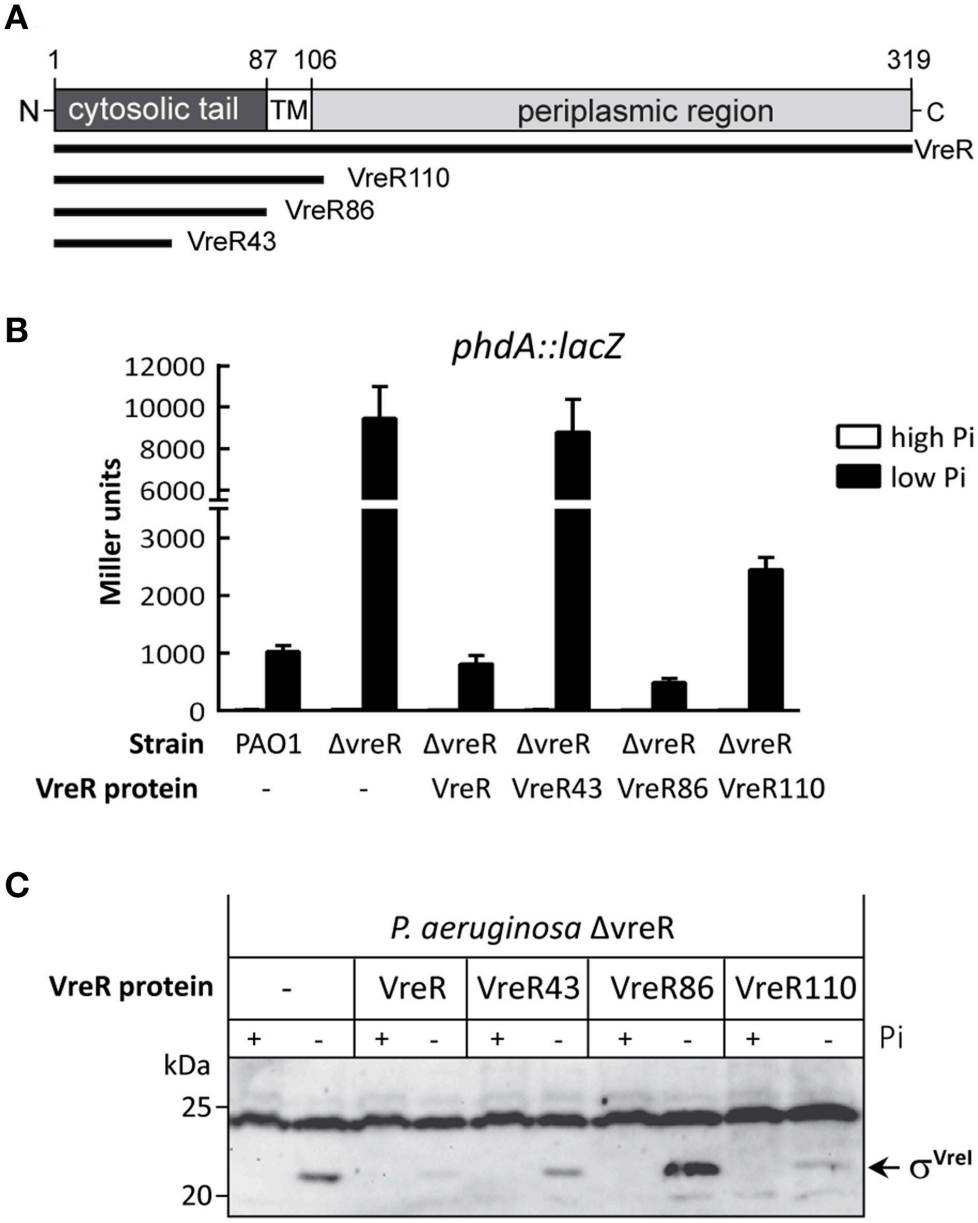
**Effect of the N-tail of VreR on σ^**VreI**^ activity. (A)** Schematic representation of the *P. aeruginosa* VreR protein. The VreR protein has been drawn to scale, and the cytosolic, transmembrane (TM), and periplasmic regions of the protein are shown. Numbers indicate amino acid positions. The produced VreR fragments are shown below the scheme. **(B)** β-galactosidase activity of the indicated *P. aeruginosa* strains bearing the transcriptional fusion *phdA*::*lacZ* and the pMMB67EH (-), pMMB-VreR, pMMB-VreR43, pMMB-VreR86 or pMMB-VreR110 plasmid expressing the indicated VreR fragment from the IPTG-inducible promoter P*tac* (Table 1). Strains were grown in high or low Pi in the presence of 1 mM IPTG. **(C)** Detection of σ^VreI^ in *P. aeruginosa* ΔvreR mutant upon expression of the indicated VreR fragment in high (+) or low (−) Pi and 1 mM IPTG. Proteins were detected by Western-blot using a polyclonal anti-VreI antibody. The positions of the molecular size marker (in kDa) and the σ^VreI^ protein are shown.

To further analyse the role of VreR in the regulation of σ^VreI^ activity, we decided to focus on the N-terminal cytosolic tail (N-tail) of VreR. This anti-σ factor fragment (about 80–90 amino acids in length) is known to bind the σ^ECF^ (Campbell et al., 2007). Although it was originally described as the domain that keeps the σ^ECF^ sequestered and inactive in absence of the inducing signal, recent data have shown that the N-tail of some anti-σ factors has pro-sigma activity and is required for σ^ECF^ functionality (Mettrick and Lamont, 2009; Bastiaansen et al., 2015). To analyse the effect of the N-tail of VreR on σ^VreI^ activity, fragments of VreR of different lengths were cloned in the pMMB67EH plasmid under the control of an IPTG-inducible P*tac* promoter (Table 1). This includes VreR43 that contains the first 43 amino acids of the VreR protein, which constitutes only half of the cytosolic N-tail; VreR86 (amino acids 1–86), which contains the entire cytoplasmic portion of VreR (the N-tail); and VreR110 (amino acids 1–110), which contains the N-tail, the transmembrane domain and 4 periplasmic residues of VreR (Figure 2A). Activity of σ^VreI^ in the presence of these protein fragments was tested in the ΔvreR mutant bearing the σ^VreI^-dependent *phdA*::*lacZ* transcriptional fusion upon growth in high and low Pi conditions. Expression of a full-length VreR protein in the mutant restored σ^VreI^ activity to wild-type levels (Figure 2B), showing that *in trans* production of VreR is able to complement the ΔvreR mutation. Amounts of σ^VreI^ in this strain were considerably lower than in the not complemented strain (Figure 2C), confirming previous observations indicating that the presence of VreR promotes σ^VreI^ degradation (Figure 1C and Llamas et al., 2009). Expression of the VreR43 fragment did not however affect σ^VreI^ activity (Figure 2B) or stability (Figure 2C), which were similar to those obtained in the not complemented ΔvreR mutant (Figure 2B). This suggests that the VreR43 fragment, which contains only half of the VreR N-tail, is unable to bind σ^VreI^. In contrast, production of the VreR86 fragment containing the complete cytosolic N-tail of VreR significantly reduced expression from the *phdA* promoter, suggesting that this fragment interacts with σ^VreI^ and inhibits its activity (Figure 2B). Expression of VreR110 also reduced σ^VreI^ activity, but to a lesser extent than VreR86 (Figure 2B), likely because the presence of the transmembrane domain in VreR110 hinders the binding of the N-tail to σ^VreI^. Interestingly, whereas expression of VreR110 results in a less stable σ^VreI^ protein when compared with the not complemented ΔvreR mutant, expression of VreR86 results in higher amounts of σ^VreI^ (Figure 2C). However, as described before, the σ factor is less active upon expression of VreR86 (Figure 2B), which implies that, in contrast to the full-length VreR and the VreR110 proteins, the VreR86-mediated inhibition of σ^VreI^ activity does not involve σ^VreI^ degradation. In accordance with the reported structures of other σ^ECF^/anti-σ pairs (Campbell et al., 2007), it is likely that the VreR86 fragment inhibits σ^VreI^ by binding to it and occluding its RNAPc binding determinants. All together these results show that overproduction of the N-tail of VreR inhibits σ^VreI^ activity, likely by interacting with this σ factor, and that therefore VreR does not contain pro-sigma activity.

### Effect of σ^VreI^ overproduction on expression of σ^VreI^-regulated genes

**Figure 3:**
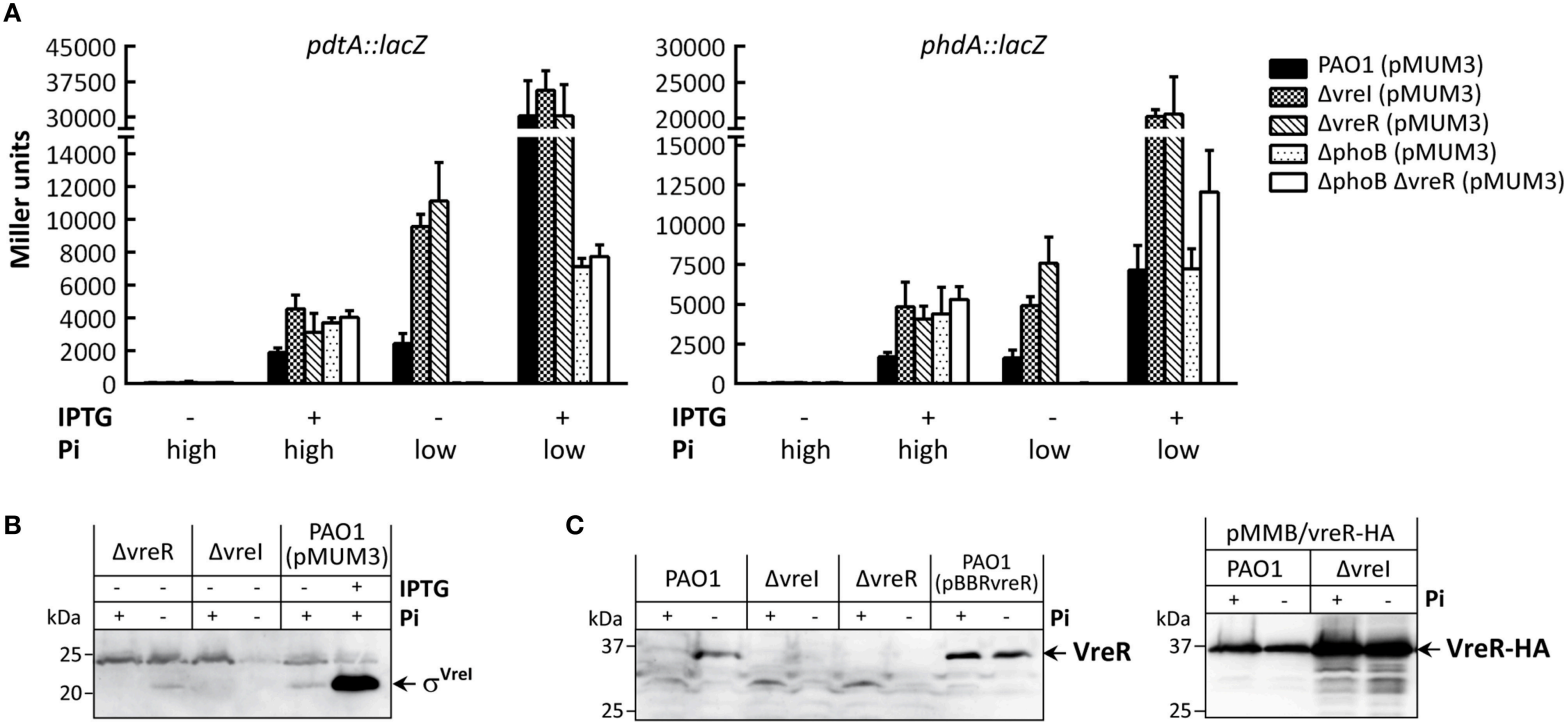
**Effect of σ^**VreI**^ overproduction in the expression of the PUMA3 regulon**. The indicated *P. aeruginosa* strains were grown 18h under high (+) or low Pi (−) conditions without (−) or with (+) 1 mM IPTG. **(A)** β-galactosidase activity of *P. aeruginosa* strains bearing the indicated *lacZ* fusion and the pMUM3 plasmid expressing the *vreI* gene from the IPTG-inducible promoter P*tac* (Llamas et al., 2009) (Table 1). **(B)** Detection of σ^VreI^ by Western-blot using a polyclonal anti-VreI antibody. The positions of the molecular size marker (in kDa) and the σ^VreI^ protein are shown. **(C)** Detection of VreR by Western-blot using a polyclonal anti-VreR antibody (left panel) or a monoclonal anti-HA antibody (right panel). The production of the VreR-HA protein from plasmid (Table 1) was induced with 1 mM IPTG. The positions of the molecular size marker (in kDa) and the VreR proteins are shown.

Several reports have shown that overproduction of σ^ECF^, including σ^VreI^, allows expression of their target genes in absence of the inducing signal (Koster et al., 1994; Pradel and Locht, 2001; Llamas et al., 2006, 2008, 2009; Faure et al., 2013). To study the effect of σ^VreI^ overproduction in the different *P. aeruginosa phoB* and PUMA3 mutants we used the pMUM3 plasmid, which contains the *vreI* gene expressed from a IPTG-inducible promoter (Llamas et al., 2009; Table 1). Activity of both P_pdtA_ and P_phdA_ was null in high Pi when expression of *vreI* from pMUM3 was not induced by IPTG (Figure 3A). Upon IPTG induction, a significant increase in activity was observed in all strains tested, including the two *phoB* mutants (Figure 3A). This effect was considerably stronger in low Pi conditions (Figure 3A). The fact that there is promoter activity in high Pi and in the *phoB* mutants when *vreI* expression is induced by IPTG indicates that overproduction of σ^VreI^ can bypass the low Pi and PhoB requirements for P_pdtA_ and P_phdA_ activity, as observed previously (Llamas et al., 2009; Faure et al., 2013). In fact, σ^VreI^ is present in extremely high amounts when its expression from pMUM3 is induced by IPTG (Figure 3B). Activity of P_pdtA_ and P_phdA_ in low Pi without IPTG was similar to that observed in low Pi in absence of the pMUM3 plasmid: Maximal in the ΔvreR single mutant and null in both *phoB* mutants (Compare Figure 3A and Figure 1B). Moreover, the ΔvreI mutant could be complemented with pMUM3 in this condition (Figure 3A, low Pi -IPTG), which suggests that σ^VreI^ is also produced from the plasmid in absence of IPTG. This was confirmed by Western-blot (Figure 3B). Interestingly, the activity of P_pdtA_ and P_phdA_ in the complemented ΔvreI mutant was considerably higher than that obtained in the PAO1 wild-type strain in low Pi without IPTG (3.9- and 3-fold higher, respectively) and similar to that of the ΔvreR mutant (Figure 3A). Since the absence of the VreR anti-σ factor results in maximal σ^VreI^ activity (Figure 1B), the observed phenotype could be due either to a polar effect of the *vreI* mutation on the expression of the downstream *vreR* gene or to the instability of the VreR protein in absence of σ^VreI^. To check these two possibilities, VreR production/stability was analyzed by Western-blot using an anti-VreR antibody that detects the chromosomally produced protein, and VreR stability was assayed using an anti-HAtag antibody that detects a C-terminally HA-tagged VreR protein constitutively produced from plasmid. This analysis showed that VreR is not produced in the ΔvreI mutant (Figure 3C, left panel), and that the stability of the protein is not affected in absence of *vreI* since even higher amount of the VreR-HA protein were detected in the ΔvreI mutant (Figure 3C, right panel). These results indicate that the *vreI* mutation exerts a polar effect on the expression of *vreR*. Therefore, both production of σ^VreI^ from pMUM3 in absence of IPTG and the lack of VreR explain the high promoter activity observed in the complemented ΔvreI mutant. Importantly, P_pdtA_ and P_phdA_ are not active in strains bearing the pMUM3 plasmid in absence of IPTG in high Pi—a condition in which PhoB is not active (Lamarche et al., 2008)—and in the two *phoB* mutants, despite the fact that σ^VreI^ is being produced and present in sufficient amount to target transcription (Figures 3A,B). This strongly suggests that PhoB is not only required for expression of the *vreI* gene but also to enhance the σ^VreI^-mediated expression of the PUMA3 regulon genes.

### Defining the promoter region of σ^VreI^-regulated genes

**Figure 4:**
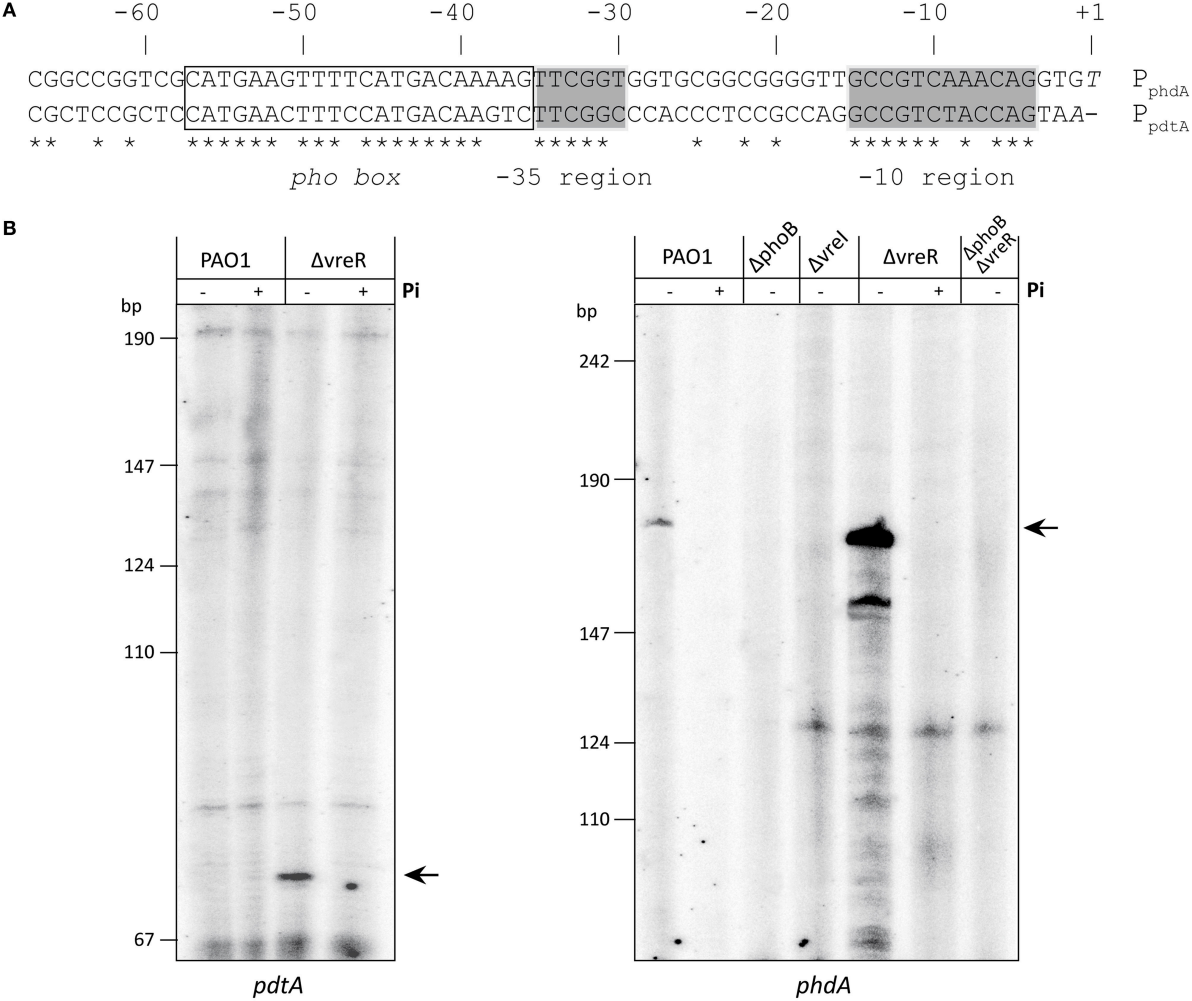
**Determination of the transcription initiation points of the ***pdtA*** and ***phdA*** genes. (A)** Identification of the +1 site by 5′ RACE and *pdtA* and *phdA* promoter analysis. The *P. aeruginosa* PAO1 genomic sequence corresponding to the region upstream of the *pdtA* and *phdA* gene is shown. Nucleotides in italic represent the proposed +1 site. The identified promoter elements (*pho*, −35 and −10 boxes) are indicated. Identical nucleotide residues in both promoter regions are marked with a star. **(B)** Primer extension analysis of *pdtA* and *phdA* mRNA. *P. aeruginosa* PAO1 cells and the indicated mutants were grown in low or high Pi medium, and samples were taken in stationary phase for total RNA isolation. The autoradiogram shows the cDNA products obtained after reverse transcription of 12 μg of total RNA with the 5′ -end-labeled PA0690R or PA0691R oligonucleotides (Table S1) hybridizing with the *pdtA* or the *phdA* mRNA, respectively.

In order to study the effect of the PhoB transcriptional regulator on the expression from P_pdtA_ and P_phdA_, we decided to first define these promoter regions by locating the transcription initiation point of the *pdtA* and *phdA* genes (Figure 1A). Transcription start sites were mapped by 5′ RACE (Invitrogen) using RNA from the *P. aeruginosa* PAO1 wild-type strain or the ΔvreR mutant after growth in low Pi medium to induce maximal *pdtA* and *phdA* expression (Figure 1B). This strategy located the transcriptional start site of *pdtA* at an adenine residue situated 53-bp upstream the *pdtA* translational start codon and that of *phdA* at a thymine residue situated 198-bp upstream the *phdA* translational start codon (Figure 4A). In order to confirm these results and to rule out the possibility of the presence of other transcription initiation points not identified by 5′ RACE, we carried out primer extension analyses. Total RNA isolated from *P. aeruginosa* PAO1 cells was annealed to a 5′ -labeled oligonucleotide complementary to either the *pdtA* or the *phdA* gene (Table S1). A single cDNA product was obtained for each gene when the RNA was isolated from *P. aeruginosa* PAO1 or ΔvreR cells grown in low Pi, the amount of these products being considerably higher in the ΔvreR mutant (Figure 4B). In fact, the *pdtA* cDNA product could be detected only in the ΔvreR mutant (Figure 4B). This confirms the maximal *lacZ* activity of the transcriptional fusions observed in ΔvreR (Figure 1B). The sizes of the cDNA products (73-bp for *pdtA* and 178-bp for *phdA*) corresponded with the transcription initiation points identified by 5′ RACE. These bands were absent when total RNA was isolated from *P. aeruginosa* cells grown in high Pi or in the ΔvreI and *phoB* mutants (Figure 4B), confirming that expression of these genes occurs under Pi starvation in a σ^VreI^- and PhoB-dependent manner (Figure 1B). An alignment of the DNA regions upstream the +1 site of *pdtA* and *phdA* genes allowed us to identify highly conserved DNA sequences centered within the −10 and −35 regions (Figure 4A). These sequences did not exhibit similarity to the consensus sequence recognized by σ^70^ (TATAAT at −10 and TTGACA at −35), and could therefore be an alternative promoter sequence recognized by the RNAP loaded with σ^VreI^. Interestingly, a putative *pho box* was detected in both promoter regions. PhoB binds DNA as a dimer and recognizes a 22-bp region with two 7-bp direct repeats followed by an A/T-rich region of 4-bp (Blanco et al., 2002), a sequence that was present in P_pdtA_ and P_phdA_ (Figure 4A). The presence of a *pho box* further suggests the direct involvement of the PhoB regulator in the expression from these promoters.

### Contribution of the −10 and −35 regions and the *pho box* to the activity of the *pdtA* promoter

To determine the contribution of the identified −10, −35 and *pho box* regions to the activity of the *pdtA* promoter, we made several constructs in which these sequences were disrupted by single or multiple substitutions (S), by insertions (I), or by deletions (Δ) (Table 2). These constructs were then fused to the *lacZ* reporter gene and transferred to the *P. aeruginosa* PAO1 wild-type strain and the ΔvreR mutant to test their activity upon growth in Pi starvation conditions. Activity of all constructs in high Pi conditions was null in both strains (data not shown), indicating that none of the mutations resulted in a constitutively active promoter. Importantly, the effect of the mutations in the promoter activity upon growth in Pi starvation was very similar when tested in the PAO1 and in the ΔvreR mutant (Table 2) in which σ^VreI^ activity is maximal, indicating that their activity depends on σ^VreI^. Single and multiple mutations in the −10 region showed that changes in the nucleotides −5 to −11 had a dramatic effect on promoter activity, which was completely abolished (Table 2). However, mutation of the −3 and −4 nucleotides had little effect (70% of the activity retained); substitution of the −12, −13, and −14 nucleotides reduced, but did not abolish the activity (30–55% of the activity retained); and mutation of only the −13 and −14 nucleotides had no effect on promoter activity (Table 2). Substitutions within the region between the −10 and −35 boxes did not affect promoter activity (Table 2; S-19 and S-24), whereas changing the size of this region by either inserting or deleting one nucleotide did significantly affect expression (Table 2; I-22 and Δ-22). Within the −35 region, substitution of the nucleotides −30 to −34 and that of the –33 and –34 considerably reduced *pdtA* promoter activity (Table 2). In contrast, changing the –29 and −30 GC nucleotides into TA resulted in a more active promoter (Table 2). The contribution of the identified *pho box* to the *pdtA* promoter activity was also analyzed. Complete disruption of the *pho box* (S pho box) or disruption of only one of the two 7-bp direct repeat sequences (S-40 to −45 or S-50 to −56) completely abolished promoter activity (Table 2). This indicates that intact −10, −35 and *pho* boxes are required for *pdtA* expression.

**Table 2:**
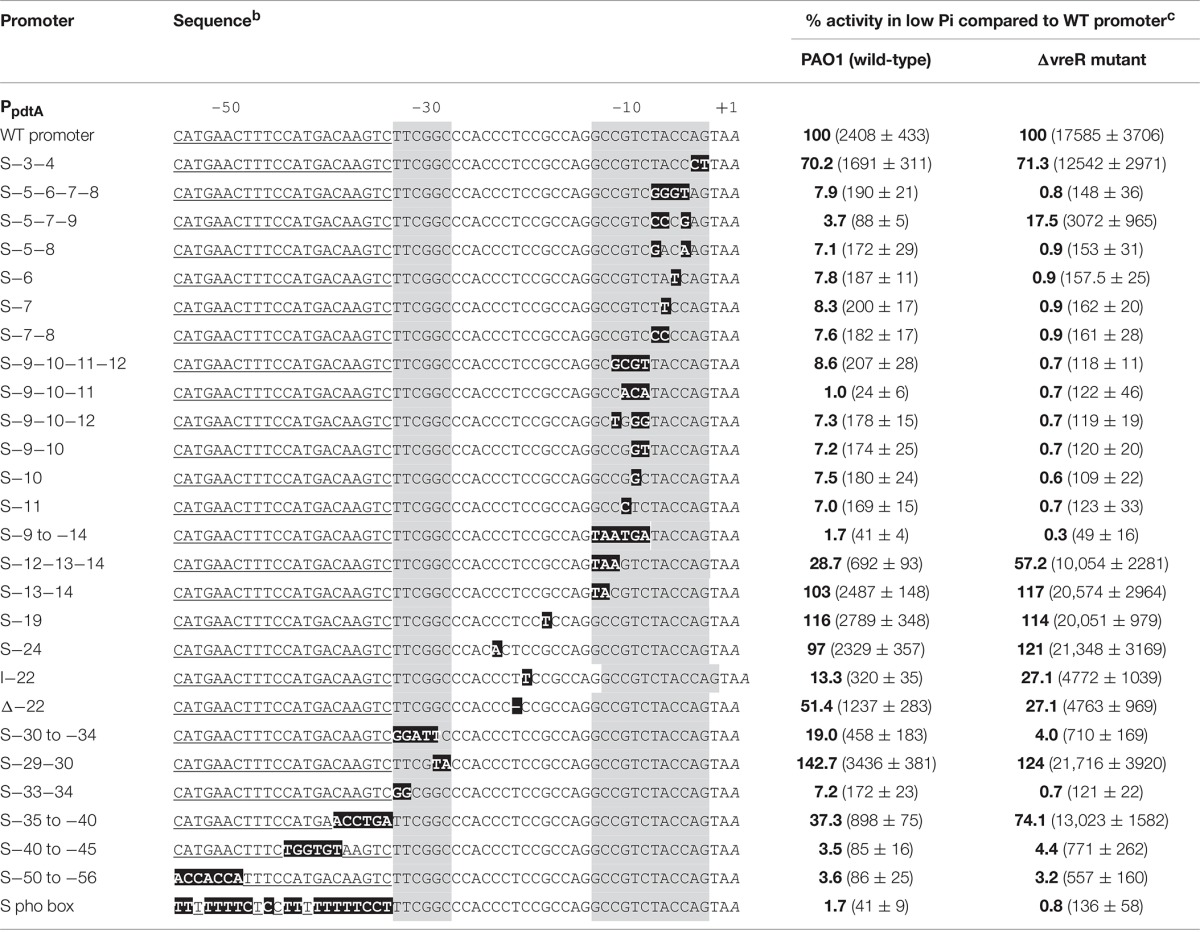
**Mutagenesis of the ***pdtA*** promoter and activity^a^**.

^a^The promoter activity was measured by β-galactosidase assay.

^b^The +1 site is in italic, the −10 and −35 regions are shaded, and the pho box is underlined.

^c^The bold values indicate % of activity compared to wild-type. Miller units and standard deviation from three biological repetitions are shown between brackets.

### The PhoB transcription factor binds to the *vreA, pdtA*, and *phdA* promoters

**Figure 5:**
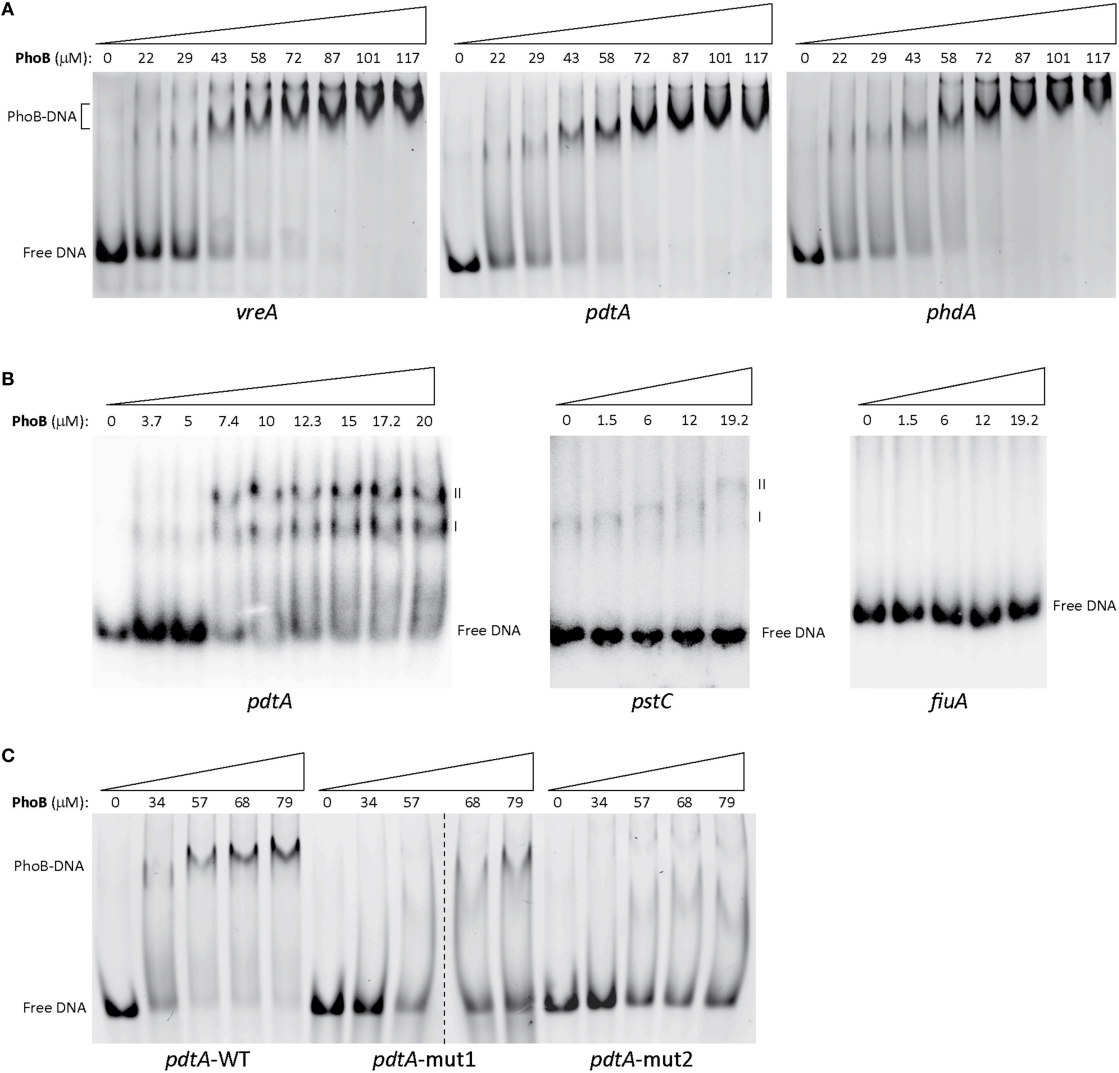
**Binding of PhoB to the ***vreA***, ***pdtA*** and ***phdA*** promoter regions**. EMSA gels using fluorescein-labeled **(A and C)** or ^32^P-labeled **(B)** dsDNA probes (Table S2) containing the indicated *P. aeruginosa* promoter and increasing amounts of phosphorylated PhoB protein. Upper numbers indicate the concentration of PhoB used in the assay (in μM). In A and B wild-type (WT) promoter sequences were used as DNA probes. In C *pdtA* promoters with mutations in the first direct repeat (*pdtA*-mut1) or in both direct repeats (*pdtA*-mut2) of the *pho box* were used. The position of the free DNA and of the PhoB-DNA complexes (I and II) are indicated.

The results obtained here with the mutational analysis of the *pho box* of the *pdtA* promoter (Table 2) and those obtained previously with a similar analysis of the *pho box* of the *vreA* promoter (Faure et al., 2013), suggest that PhoB directly binds to these promoter regions. To confirm this, we performed electrophoretic mobility shift assays (EMSA) using a fixed amount of fluorescein-labeled dsDNA probes obtained by annealing oligonucleotides that contain the promoter region of the *vreA, pdtA*, or *phdA* genes (Table S2). Addition of increasing concentrations of purified and phosphorylated PhoB protein to the DNA fragments resulted in a slower complex that at higher protein concentration became the predominant (Figure 5A), showing that PhoB indeed binds the *vreA, pdtA* and *phdA* promoters. Since non-isotopic DNA labeling can alter the affinity and/or stoichiometry of the protein-DNA interaction, we also performed the EMSA using ^32^P-labeled dsDNA. In this condition, two retarded DNA bands were observed (Figure 5B, *pdtA* promoter). Since PhoB is known to bind to DNA as a dimer of which each monomer contacts one direct repeat of the *pho box* (Makino et al., 1996; Blanco et al., 2002), it is possible that these bands are the result of PhoB binding first as a monomer (complex I) and at higher concentrations as a dimer, generating the second retardation band (complex II). Two DNA retardation bands were also observed when the *pstC* promoter, which is known to contain a *pho box* (Nikata et al., 1996; Jensen et al., 2006), was used as a positive control (Figure S2). No band shifts were however detected when the *fiuA* promoter, which is not regulated by low Pi (Llamas et al., 2006) and does not have a *pho box*, was used as DNA probe (Figure S2). This confirms the specific binding of the purified PhoB protein to promoters containing a *pho box*. Moreover, addition of increasing amounts of an unlabeled competitor dsDNA to the EMSA reactions resulted in the complete disappearance of the second retardation band and in a considerably increase in the amount of free DNA (Figure S2). Although the complex I was still formed (probably because the amounts of unlabeled DNA did not reach the level needed for complex I to disappear), this indicates that there is competition between the DNAs and therefore that the retardation bands are the specific result of PhoB-DNA complexes formation. Interestingly, when a *pdtA* promoter containing mutations in the first direct repeat of the *pho box* was used as DNA probe, the binding of PhoB was considerably impaired and a higher concentration of the protein was needed for the formation of the PhoB-DNA complex (Figure 5C). Mutation of the two direct repeats of the *pho box* completely abolished PhoB binding (Figure 5C), which suggests that this mutated region contains the PhoB binding site. Altogether, our results show that PhoB binds to the promoter region of the *vreAIR* operon and, importantly, to that of the σ^VreI^-dependent promoters *pdtA* and *phdA*.

### PhoB is required for the binding of σ^VreI^ to the *pdtA* promoter

**Figure 6:**
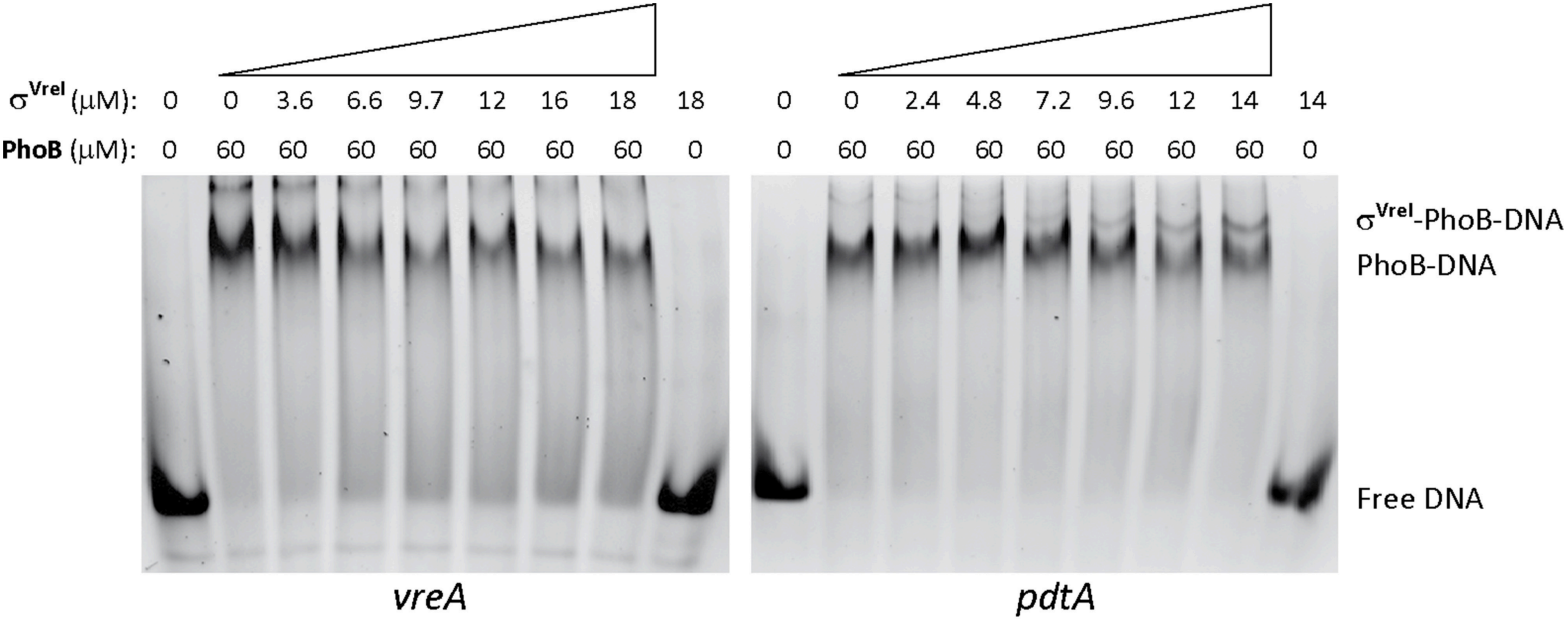
**Binding of σ^**VreI**^ to the ***pdtA*** promoter**. EMSA gels using fluorescein-labeled dsDNA probes containing the *P. aeruginosa vreA* or *pdtA* promoter (Table S2). Increasing amounts of σ^VreI^ were added to a preformed PhoB-DNA complex. Upper numbers indicate the concentration of phosphorylated PhoB and σ^VreI^ proteins used in the assay (in μM). The position of the free DNA and of the PhoB-DNA and σ^VreI^-PhoB-DNA complexes are indicated.

Next, we assayed the binding of σ^VreI^ to the *pdtA* promoter by EMSA. Several attempts using the σ^VreI^ protein alone or reconstituted σ^VreI^-RNAP holoenzyme did not result in DNA retardation bands (data not shown), suggesting that σ^VreI^ alone could not bind to this promoter region. Therefore, we tested the binding of σ^VreI^ to PhoB-DNA complexes. Addition of increasing amounts of σ^VreI^ in EMSA reactions containing the PhoB protein (in a concentration that results in the formation of the complex II) and the *pdtA* promoter resulted in the appearance of a new retarded DNA band (Figure 6). This change in mobility likely reflects the formation of a σ^VreI^-PhoB-DNA complex, which was not formed in absence of σ^VreI^ or PhoB (Figure 6). This indicates that σ^VreI^ cannot interact with the promoter region of *pdtA* and *phdA* genes in the absence of PhoB, which is in agreement with the PhoB-requirement for expression of these genes even in conditions in which σ^VreI^ is present (Figure 3A, low Pi -IPTG). The σ^VreI^-PhoB-DNA complex was not formed when the *vreA* promoter was used as DNA probe (Figure 6), in agreement with σ^VreI^ not being involved in the expression from this promoter (Figure 1B). This confirms the specific binding of the σ factor to σ^VreI^-dependent promoters and shows the requirement of PhoB for this binding to occur.

## Discussion

The PUMA3 system of *P. aeruginosa* is an unusual CSS cascade in various functional and architectural aspects. Importantly, this signal transduction system is directly involved in the regulation of virulence and, unlike most *P. aeruginosa* CSS systems, does not control iron uptake (Llamas et al., 2009, 2014). The architectural variations of the system mainly concern the VreA receptor-like component, which is smaller than regular CSS receptors and seems to function only in signaling and not in the transport of the signal molecule (Llamas et al., 2009). In addition, the genetic organization of the *vreAIR* genes encoding the PUMA3 system is different than that of most CSS pathways. While CSS σ^ECF^ are generally co-transcribed with their cognate anti-σ factor and the receptor gene is located in a separate transcriptional unit (Koebnik, 2005; Llamas et al., 2014), the *vreAIR* genes form an operon (Figure 1A). In *P. aeruginosa* expression of most σ^ECF^/anti-σ operons is controlled by iron through the Fur regulator, which allows production of these proteins in iron depleted conditions (Llamas et al., 2014). In contrast, expression of the *vreAIR* operon is targeted by Pi starvation and requires the phosphate regulator PhoB. A *pho box* is present in the *vreA* promoter region (Faure et al., 2013), and direct binding of this transcription factor to this promoter region has been demonstrated in this work (Figure 5). The *vreAIR* gene products, including σ^VreI^, are not involved in the expression from the *vreA* promoter, and in accordance, σ^VreI^ does not bind to this promoter (Figure 6). This indicates that another σ factor, likely the *P. aeruginosa* primary σ factor σ^70^, targets transcription of the *vreAIR* operon under Pi starvation and in a PhoB-dependent manner.

Interestingly, the genes belonging to the PUMA3 regulon are expressed in response to Pi starvation in a σ^VreI^-dependent manner, despite the fact that in this condition the σ^VreI^ repressor VreR is also produced. A specific inducing signal is typically required to relieve the anti-σ-mediated inhibition of σ^ECF^ activity. In presence of such a signal, the anti-σ factor protein is removed by regulated proteolysis allowing the σ^ECF^-mediated transcription (Qiu et al., 2007; Ades, 2008; Draper et al., 2011; Bastiaansen et al., 2014, 2015). We show here that deletion of the *vreR* anti-σ factor gene is required for maximal σ^VreI^ activity in low Pi (Figure 1B). This suggests that an additional stimulus not present in Pi starvation is needed to remove VreR and produce full σ^VreI^ activation. This hypothesis is supported by the fact that the VreA receptor, which by analogy with most CSS systems likely initiates the PUMA3 signaling cascade (Llamas et al., 2014), is not required for the transcription of the PUMA3 regulon genes in low Pi. Therefore, the detected σ^VreI^ activity in Pi starvation seems not to be the result of actual signaling through the PUMA3 CSS system, but represents “leaky” activity of σ^VreI^. Previous studies have shown that σ^VreI^-regulated genes are induced upon contact of *P. aeruginosa* with human airway epithelial cells (Frisk et al., 2004; Chugani and Greenberg, 2007). Moreover, antibodies against the PdtA and PA0697 proteins of the PUMA3 regulon (Figure 1A) have been detected in the serum of patients infected with *P. aeruginosa* (Llamas et al., 2009). This suggests that the molecule targeting PUMA3 signaling could be host-derived, and our current work aims at identifying such a signal.

The fact that VreR removal produces maximal activation of σ^VreI^ indicates that this anti-σ factor only has anti-σ function. Two divergent classes of CSS anti-σ factors have been reported, mere anti-σ factors and anti-σ factors with pro-sigma activity (also called sigma factor regulators) (Mettrick and Lamont, 2009; Llamas and Bitter, 2010; Llamas et al., 2014). Proteins within the first group only contain anti-σ activity and inhibit activity of their cognate σ^ECF^ in absence of the CSS inducing signal. Deletion of these proteins results in signal-independent transcription of the σ^ECF^-regulated genes (Mettrick and Lamont, 2009). In contrast, deletion of anti-σ factors of the second group does not result in activation of its cognate σ^ECF^ since these anti-σ factors are required for σ^ECF^ activity. The pro-sigma activity of these proteins seems to reside within the short cytosolic N-terminal region (N-tail), since the expression of this domain alone induces σ^ECF^ activity independently of the presence of the signal (Ochs et al., 1995; Ó Cuív et al., 2006; Mettrick and Lamont, 2009). Recently, we have shown that the N-tail of anti-σ factors is indeed produced *in vivo* in response to the inducing signal and that the transmembrane protease RseP is responsible for this process (Bastiaansen et al., 2015). Although still not experimentally determined, it has been proposed that the N-tail can protect the σ^ECF^ from degradation and that this domain may be bound to the σ^ECF^-RNAP holoenzyme during the transcription process (Mahren and Braun, 2003). However, this does not seem to be the case for the N-tail of VreR since this protein fragment does not enhance σ^VreI^ activity. In fact, overexpression of the N-tail of VreR inhibits the activity of σ^VreI^ (Figure 2). This indicates that VreR does not contain pro-sigma activity, which is in accordance with the higher σ^VreI^ activity detected in the ΔvreR mutant. The role of VreR as a mere anti-σ factor is further supported by the fact that σ^VreI^ is more stable in absence of VreR. Our results suggest that VreR employs at least two mechanisms to inhibit σ^VreI^ activity: binding to the σ factor likely shielding the binding determinants of σ^VreI^ for the RNAPc, and promotion of σ^VreI^ degradation. The N-tail of VreR (aminoacids 1–86) seems to be sufficient to prevent binding of σ^VreI^ to the RNAPc, but this fragment alone does not promote σ^VreI^ degradation (Figure 2) and the complete protein seems to be required for this. Another *P. aeruginosa* CSS anti-σ factor, FvpR, has also been reported to induce degradation of its cognate σ^ECF^ (Spencer et al., 2008), although the mechanism behind this process is still unknown. These observations further indicate that *in vivo* and upon sensing the PUMA3 inducing signal, VreR needs to be completely removed in order for σ^VreI^ to reach maximal activity.

Importantly, we show in this work that activity of σ^VreI^ is also modulated by a transcription factor, the phosphate regulator PhoB. This is an important finding since, while modulation of primary σ factors activity by trans-acting factors has been extensively reported, such modulation of σ^ECF^ has not been extensively investigated yet. As demonstrated in this study, PhoB is not only required for σ^VreI^ production but also for the binding of σ^VreI^ to the promoter region of its target genes. In fact, the two proteins bind to the promoter of the σ^VreI^-regulated genes and, in accordance, expression of these genes does not occur unless both proteins are present and active in the cell. Only extremely high levels of σ^VreI^, which we obtained by overexpressing the *vreI* gene from an IPTG-inducible promoter, can bypass the PhoB requirement for the transcription of the σ^VreI^ target genes. However, these σ^VreI^ levels are not likely to be ever reached *in vivo*. As mentioned before, it is expected that upon sensing the PUMA3 inducing signal VreR is proteolytically degraded and σ^VreI^ released. Thus, the maximal σ^VreI^ amount expected *in vivo* upon induction of the PUMA3 cascade likely resembles the level obtained in the ΔvreR mutant, which is considerably lower than that obtained when production of σ^VreI^ from plasmid was induced with IPTG (Figure 3B). Therefore, both PhoB and σ^VreI^ are needed to target transcription of the PUMA3 regulon genes *in vivo*. The potential DNA binding sites for PhoB and σ^VreI^ in the promoter regions of PUMA3 regulon genes have been identified. A conserved *pho box* (Blanco et al., 2002) containing two 7-bp direct repeats is located upstream of the −35 region of both the *pdtA* and *phdA* promoters. Mutation of this region, either one of the direct repeats or the entire box, completely abrogates gene expression (Table 2), and, when the two direct repeats are mutated, also the binding of PhoB (Figure 5C). Based on these results, we propose that this region within the *pdtA* and *pdhA* promoters is the PhoB binding site. Although in *E. coli* the *pho box* is usually located near the σ^70^ –10 promoter region substituting the −35 region (Makino et al., 1986; Blanco et al., 2002), this does not seem to be the case for σ^VreI^-dependent promoters. Downstream of the *pho box*, highly identical sequences centered within the −35 and −10 positions have been identified in the *pdtA* and *phdA* promoters (Figure 4A). Members of the σ^70^ family are known to recognize promoter sequences located at positions −35 and −10 from the transcriptional start point and regions 4.2 and 2.4, respectively, of the primary σ^70^ protein are involved in such recognition (Brooks and Buchanan, 2008). σ^ECF^ are the smallest σ factors of the σ^70^ family and lack two of the four conserved domains of primary σ factors (domains 1 and 3) (Lonetto et al., 1994; Bastiaansen et al., 2012). However, promoter recognition by σ^ECF^ involves the same σ factor regions (Enz et al., 2003; Wilson and Lamont, 2006). Interestingly, region 2.4, which recognizes the −10 promoter element, shows most variation within the σ^ECF^ subfamily, which likely reflects differences in promoter binding specificity (Lonetto et al., 1994). This suggests that promoter specificity of σ^ECF^ is predominantly determined by the −10 promoter element and the region 2.4 of the σ^ECF^. In agreement, single mutations within the −10 promoter sequence of *pdtA* (nucleotides −5 to −11) completely abrogated gene expression, in both the wild-type strain and the ΔvreR mutant, which strongly indicates that this region is essential for the σ^VreI^-mediated transcription of this gene. Although σ^ECF^ usually share a high degree of similarity in their −35 promoter element (Enz et al., 2003), which has a conserved AA motif that is important for DNA geometry and thus for σ^ECF^-DNA interaction (Lane and Darst, 2006), this is not the case for the σ^VreI^-dependent promoters. The absence of this motif in the −35 region could impair the binding of σ^VreI^ to the DNA, which would be facilitated by the binding of the PhoB protein to the *pho box*. Our results strongly suggest a model in which PhoB recruits σ^VreI^ to the promoter region to trigger transcription, which is similar to the mechanism employs by PhoB with the primary σ^70^ factor (Makino et al., 1996; Blanco et al., 2011). Although studies focused on the structure of the PhoB-σ^VreI^-DNA complex are required to fully understand the process, it is likely that the PhoB-σ^VreI^ interaction involves, as shown for σ^70^ (Blanco et al., 2011), the region 4 of σ^VreI^, which is the region that contacts the −35 sequence and is potentially the closest to PhoB in the complex.

In summary, our results show that the activity of the *P. aeruginosa* σ^VreI^ in Pi starvation is modulated by both the anti-σ factor VreR and the transcription factor PhoB. Pi starvation is an important environmental cue that induces transcription of the so-called *pho* regulon, which in *P. aeruginosa* includes multiple potential virulence factors (Lamarche et al., 2008). It is therefore not surprising that Pi starvation enhances *P. aeruginosa* lethality in mice and nematodes, while providing excess phosphate protects from killing (Long et al., 2008; Zaborina et al., 2008; Zaborin et al., 2009, 2012). Overexpression of σ^VreI^ has been demonstrated to increase *P. aeruginosa* lethality in zebrafish embryos (Llamas et al., 2009) and preliminary results from our group indicate that Pi starvation enhances the virulence of this bacterium in this infection model (data not shown). Moreover, there are several indications that the *P. aeruginosa pho* regulon is induced *in vivo* during infection (Frisk et al., 2004; Datta et al., 2007; Long et al., 2008). Since the PUMA3 CSS system is produced under Pi starvation and the currently unknown inducing signal is likely host-derived, it will be of interest to determine the contribution of σ^VreI^ and the PUMA3 regulon proteins to the low Pi-induced virulence of *P. aeruginosa*.

## Author contributions

JQ and ML conceived and designed the study. JQ, JO, KB, and CC performed the experiments. JQ, KB, and ML analyzed and interpreted the data. ML wrote the manuscript.

## Funding

This work has been supported by the EU Seventh Framework Programme through a Marie Curie CIG grant (3038130), and the Spanish Ministry of Economy with grants inside the Ramón&Cajal (RYC2011-08874 to ML) and the Plan Nacional for I+D+i (SAF2012-31919 and SAF2015-68873-P) programs.

### Conflict of interest statement

The authors declare that the research was conducted in the absence of any commercial or financial relationships that could be construed as a potential conflict of interest.

## Abbreviations

CSS: cell-surface signaling
ECF: extracytoplasmic function
IPTG: isopropyl β-D-1-thiogalactopyranoside
RNAPc: RNA polymerase core enzyme
Pi: inorganic phosphate.
