# Supplementary material for: The Activity of the Pseudomonas aeruginosa Virulence Regulator σVreI Is Modulated by the Anti-σ Factor VreR and the Transcription Factor PhoB
Source: Front Microbiol. 2016 Aug 3;7:1159. doi: 10.3389/fmicb.2016.01159 (PMC4971064; doi:10.3389/fmicb.2016.01159)
Supplement: Supplementary file 1 [file DataSheet1.PDF]

Supplementary Material

The activity of the *Pseudomonas aeruginosa* virulence regulator σ^{VreI} is modulated by the anti-σ factor VreR and the transcription factor PhoB

Jose M. Quesada, Joaquín R. Otero-Asman, Karlijn C. Bastiaansen, Cristina Civantos, and María A. Llamas*

*** Correspondence:** marian.llamas@eez.csic.es

Supplementary Figure 1

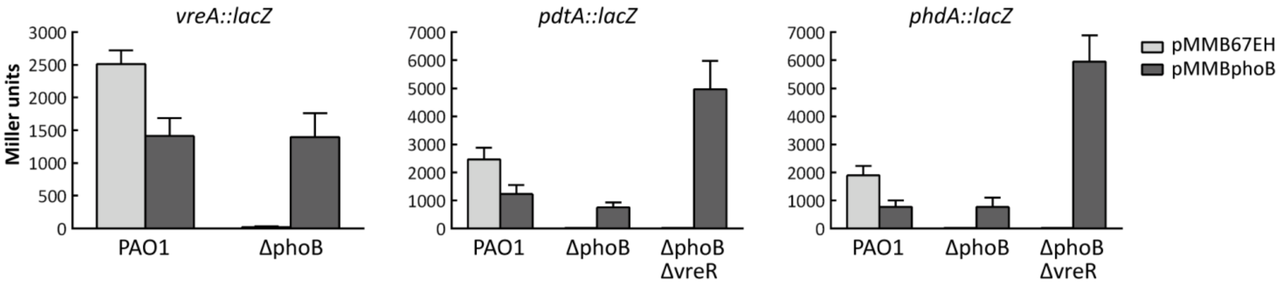

Figure S1. Complementation of the *P. aeruginosa phoB* mutants. β-galactosidase activity of the indicated *P. aeruginosa* strains bearing the *vreA*, *pdtA* or *phdA lacZ* transcriptional fusion and the pMMB67EH (empty) or pMMBphoB (containing the *phoB* gene) plasmids upon growth in Pi starvation conditions.

Supplementary Figure 2

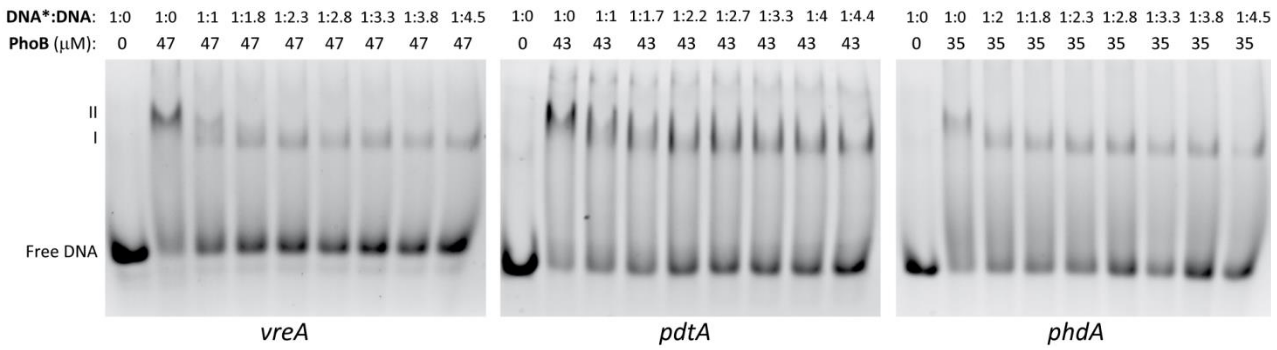

Figure S2. Competition EMSA reactions with unlabelled probes. EMSA gels using fluorescein-labelled dsDNA probes and increasing amounts of an unlabelled competitor (Table S2) containing the indicated *P. aeruginosa* promoter. Upper numbers indicate the ratio of labelled-DNA (*) and unlabelled-DNA, and the concentration of phosphorylated PhoB protein used in the assay (in µM). The position of the free DNA and of the PhoB-DNA complexes (I and II) are indicated.

Table S1. Sequence of the primers used in this study

Amplified (or deleted) gene and promoter region	Plasmid/assay	Name	Sequence (5’ → 3’)[a]
vreA (PA0674)	pKΔvreA	PA0673F-X	CAT**TCTAGA**CAACCTGCTGACCAACGAG
		ΔPUMA3R-E	CCA**GAATTC**ATTCCAGTCCGAACTACCC
		vreIF-E	GAT**GAATTC**TGACGGAGGGAGTGGGAGGG
		C-VreRR-B	AAT**GGATCC**TCAGCCGAGCAGCACCACC
vreI (PA0675)	pET-vreI	VreIF-Nd	ACA**CATATG**AGCGATTCGCGGCAAAGC
		PA0675R-B	TAT**GGATCC**CCTGCTTATGCTTATGACGG
vreR (PA0676)	pKΔvreR	pUCMA3F-X	TCA**TCTAGA**GTCCACTCGTCCG
		ΔvreRR-E	ACT**GAATTC**GACTGTGCTGTACGGACAC
		ΔvreRF-E	AAA**GAATTC**GGGGTGGTGCTGCTCGGC
		PA0678R-B	TTC**GGATCC**ATCTTCGTCGGGCACTCTG
	pMMB-VreR and pBBRvreR	PA0676F_4-Kp	GAAT**GGTACC**ATGACAGCCTCAGACTCCGC
		PA0676R_960-H	ACAC**AAGCTT**TCAGCCGAGCAGCACCACC
	pMMB-VreR43	PA0676F_4-Kp	GAAT**GGTACC**ATGACAGCCTCAGACTCCGC
		PA0676R_43-H	ACA**AAGCTT**ACGCACACCACTGGCGGAAGG
	pMMB-VreR86	PA0676F_4-Kp	GAAT**GGTACC**ATGACAGCCTCAGACTCCGC
		PA0676R_85-H	TTA**AAGCTT**AGCGACGACCGAAACGCCTC
	pMMB-VreR110	PA0676F_4-Kp	GAAT**GGTACC**ATGACAGCCTCAGACTCCGC
		PA0676R_110-H	TTA**AAGCTT**AGCCATGGTCGAGCAGCGACG
	pMMB/VreR-HA	PA0676Fa-E	AAA**GAATTC**ATGACAGCCTCAGACTCCGCCGCC
		CHA-PA0676-X	TTT**TCTAGA**TTAGCACGCGTAGTCCGGCACGTC GTACGGGTAGCCGAGCAGCACCACCCCGCCCGG
pdtA (PA0690)	pMP0690	PR0690F-E	TTA**GAATTC**TCATGAGCGCCTTCATCACTGGTA
		PR0690R-X	TAA**TCTAGA**AACGCTGCAACTGCTGGTTGA
	pTOPO-Pr0690	PR0690 F-E	TTA**GAATTC**TCATGAGCGCCTTCATCACTGGTA
		MCS220R	ATCAACGGTGGTATATCC
	5’ RACE	GSP1B-0690	GAACATCCTGCGGGAGATTC

		GSP2-0690	TGCTGGTTGACCCGCTGCTGCT
	Primer extension	PA0690R	GACGGCGAACGGGACGGCA
phdA (PA0691)	pTOPO-Pr0691b	PR0691F2Bg	TAA**AGATCT**GATCGCCGCGCTGTTCCCCGAG
		MCS220R	ATCAACGGTGGTATATCC
	5' RACE	GSP1-0691	CCGACGAACCAGCGATACAG
		GSP2-0691	CGCCCCCTGCCAATCTTCCTG
	Primer extension	PA0691R	CGTATTCACCGGAGTGACCA
phoB (PA5360)	pET-phoB	PhoBF-Nd	TAG**CATATG**GTTGGCAAGACAATCCTC
		PhoBR-B	AAC**GGATCC**TCAGCTCTTGGTGGAGAAACG
	pMMB-phoB	1147F-E	AGAG**GAATTC**AACCTGTTGAGCATAGCTC
		1148R-H	AGAG**AAGCTT**TCAGCTCTTGGTGGAGAAAC

[a] The sequences of the restriction sites are indicated in bold

Table S2. Oligonucleotides used in the EMSA reactions

Name	Sequence (5' → 3')[a]
Flu-PvreAF	[Flc]-ACCGCAGGTACCGTCACACCACAGTCACACAGTGCCATCAGGATGTCCCTCCTGGGTGATGGCCATCTGG
PvreAF	ACCGCAGGTACCGTCACACCACAGTCACACAGTGCCATCAGGATGTCCCTCCTGGGTGATGGCCATCTGG
PvreAR	CCAGATGGCCATCACCCAGGAGGGACATCCTGATGGCACTGTGTGACTGTGGTGTGACGGTACCTGCGGT
Flu-PpdtAF	[Flc]-CGCTCCGCTCCATGAACTTTCCATGACAAGTCTTCGGCCCACCCTCCGCCAGGCCGTCTACCAGTAA
PpdtAF	CGCTCCGCTCCATGAACTTTCCATGACAAGTCTTCGGCCCACCCTCCGCCAGGCCGTCTACCAGTAA
PpdtAR	TTACTGGTAGACGGCCTGGCGGAGGGTGGGCCGAAGACTTGTCATGGAAAGTTCATGGAGCGGAGCG
Flu-PpdtAmutF1	[Flc]-CGCTCCGCTC**ACCACCA**TTTCCATGACAAGTCTTCGGCCCACCCTCCGCCAGGCCGTCTACCAGTAA
PpdtAmutR1	TTACTGGTAGACGGCCTGGCGGAGGGTGGGCCGAAGACTTGTCATGGAAATGGTGGTGAGCGGAGCG
Flu-PpdtAmutF2	[Flc]-CGCTCCGCTC**ACCACCA**TTTC**TGGTGT**AAGTCTTCGGCCCACCCTCCGCCAGGCCGTCTACCAGTAA
PpdtAmutR2	TTACTGGTAGACGGCCTGGCGGAGGGTGGGCCGAAGACTTACACCAGAAATGGTGGTGAGCGGAGCG
Flu-PphdAF	[Flc]-CGGCCGGTCGCATGAAGTTTTCATGACAAAAGTTCGGTGGTGCGGCGGGGTTGCCGTCAAACAGGTGT
PphdAF	CGGCCGGTCGCATGAAGTTTTCATGACAAAAGTTCGGTGGTGCGGCGGGGTTGCCGTCAAACAGGTGT
PphdAR	ACACCTGTTTGACGGCAACCCCGCCGCACCACCGAACTTTTGTCATGAAAACTTCATGCGACCGGCCG

[a]Flc indicates fluorescein. The *pho box* is underlined and nucleotide substitutions relative to the wild-type sequence are in bold.
